# Circulating adiponectin levels in systemic sclerosis: A meta-analysis and bidirectional Mendelian randomization study

**DOI:** 10.1177/23971983251352341

**Published:** 2025-07-17

**Authors:** Tahzeeb Fatima, Cecilia Överdahl, Cristina Maglio

**Affiliations:** 1 Department of Rheumatology and Inflammation Research, Institute of Medicine, Sahlgrenska Academy, University of Gothenburg, Gothenburg, Sweden; 2 Rheumatology Clinic, Sahlgrenska University Hospital, Gothenburg, Sweden

**Keywords:** Systemic sclerosis, adiponectin, meta-analysis, Mendelian randomization, diffuse cutaneous, limited cutaneous

## Abstract

**Background:**

Previous data on the relationship between adiponectin and systemic sclerosis are inconsistent and do not establish a causal link. We aimed to perform an updated meta-analysis to estimate the association between circulating adiponectin and systemic sclerosis. Using a two-sample, bidirectional, Mendelian randomization approach, we also tested for a causal relationship between genetically predicted adiponectin levels and systemic sclerosis risk.

**Methods:**

We conducted a systematic literature search of PubMed, Embase, and Web of Science (up to September 2024) to identify studies for meta-analysis. Pooled standardized mean differences were calculated. For bidirectional Mendelian randomization, genetic instruments for circulating adiponectin levels and liability to systemic sclerosis were constructed using publicly available genome-wide association study summary statistics. Causal estimates were primarily summarized using the inverse variance-weighted method, with weighted median, simple median, MR-Egger, and MR-PRESSO as sensitivity analyses. Both meta- and Mendelian randomization analyses were stratified for systemic sclerosis subtypes: diffuse cutaneous and limited cutaneous systemic sclerosis.

**Results:**

Seven studies (439 systemic sclerosis cases, 274 controls) were included in the meta-analysis, indicating lower circulating adiponectin levels in systemic sclerosis patients (standardized mean difference = −0.16, *p* = 0.07); however, the decrease was statistically significant in diffuse cutaneous systemic sclerosis (*p* = 0.003) but not limited cutaneous systemic sclerosis (*p* = 0.81) subgroup. Forward Mendelian randomization analysis did not suggest a causal effect of adiponectin on systemic sclerosis risk (odds ratio = 1.21, *p* = 0.57), whereas reverse Mendelian randomization provided evidence for a causal effect of genetic liability to systemic sclerosis on lowering circulating adiponectin levels (ß = −0.027, *p* = 6.8E-06).

**Conclusion:**

Our meta-analysis of observational studies confirmed that systemic sclerosis patients have lower adiponectin levels. Using Mendelian randomization, we established a causal link between genetic liability to systemic sclerosis and lower adiponectin levels. These findings, limited to European ancestry, warrant further research to explore the relationship between systemic sclerosis and adiponectin levels in diverse populations.

## Introduction

Systemic sclerosis (SSc) is a chronic systemic rheumatic disease, characterized by vasculopathy, activation of the immune system with autoantibody production, and fibrosis of the skin and often of internal organs leading to a wide range of symptoms and manifestations.^1^ Based on the degree of skin involvement, SSc is classified into diffuse cutaneous (dc) and limited cutaneous (lc) subtypes.^1,2^ The prevalence of the disease is higher in females compared to males, while in total it is estimated to affect between 150 and 300 individuals per million in Europe.^3^ SSc etiology is largely unknown, where both environmental and genetic factors are thought to play a role in the disease initiation.^4,5^

Adiponectin, a cytokine secreted by adipose tissue, plays a key role in regulating insulin sensitivity, energy metabolism, inflammation and autoimmunity.^6-9^ Adiponectin has been suggested to be involved in the pathogenesis of SSc by virtue of its anti-fibrotic features and a possible effect on inflammatory reactions.^10^ It has been shown to attenuate TGF-β1-induced fibroblast activation and extracellular matrix production ^11^ and to suppress pro-inflammatory cytokines such as IL-6 and TNF-α.^12^ Notably, reduced adiponectin pathway activity and receptor expression have been observed in SSc skin biopsies.^13^ Previously, two meta-analyses of observational studies have reported lower circulating adiponectin in SSc patients compared to healthy controls. However, their results did not reach statistical significance in Europeans when ethnicity-stratified analyses were performed.^14,15^ Since then, several studies in Europeans have been conducted, with two reporting lower serum levels of adiponectin in SSc patients compared to controls,^16,17^ while the others found no significant difference.^18-20^

Mendelian randomization (MR) is an analytical approach that uses genetic variants associated with an exposure to ascertain a causal relationship with an outcome, while accounting for confounding and reverse causality.^21,22^ Despite the findings from the observational studies, no published research has yet investigated a causal link between circulating adiponectin levels and the risk of SSc. Here, we performed an updated meta-analysis of observational studies to derive a more accurate estimation of the association between circulating adiponectin and SSc. The analysis was extended to test for a causal relationship between genetically predicted adiponectin levels and SSc risk using a two-sample, bidirectional, MR approach. All analyses were carried out in European individuals.

## Methods

**Figure 1.**
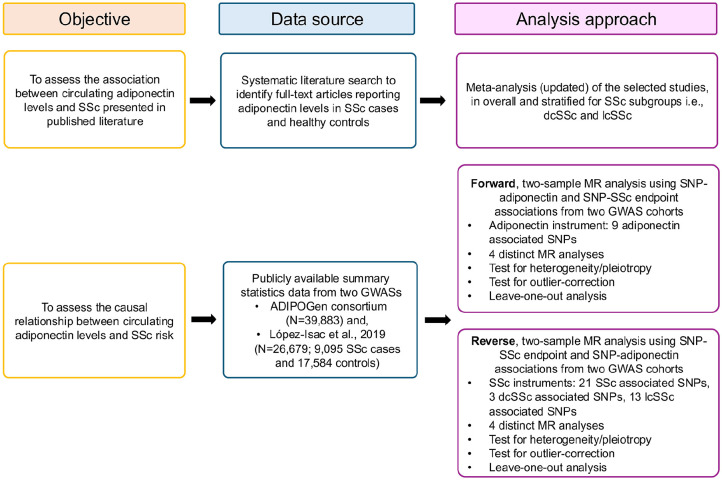
An overview of the study design. SSc: Systemic sclerosis, dcSSc: diffuse cutaneous systemic sclerosis, lcSSc: limited cutaneous systemic sclerosis, *N*: number of participants, MR: Mendelian randomization, SNP: single nucleotide polymorphism/variant, GWAS: genome-wide association study.

Figure 1 provides an overview of the study design, which consisted of two main components—a meta-analysis and a causal association (or MR) analysis—used to comprehensively assess the relationship between circulating adiponectin levels and SSc risk in people of European/Caucasian ancestry.

### Meta-analysis

The meta-analysis in this study was conducted in accordance with the Preferred Reporting Items for Systematic Reviews and Meta-Analyses (PRISMA) guidelines.^23^

### Publication search

**Figure 2.**
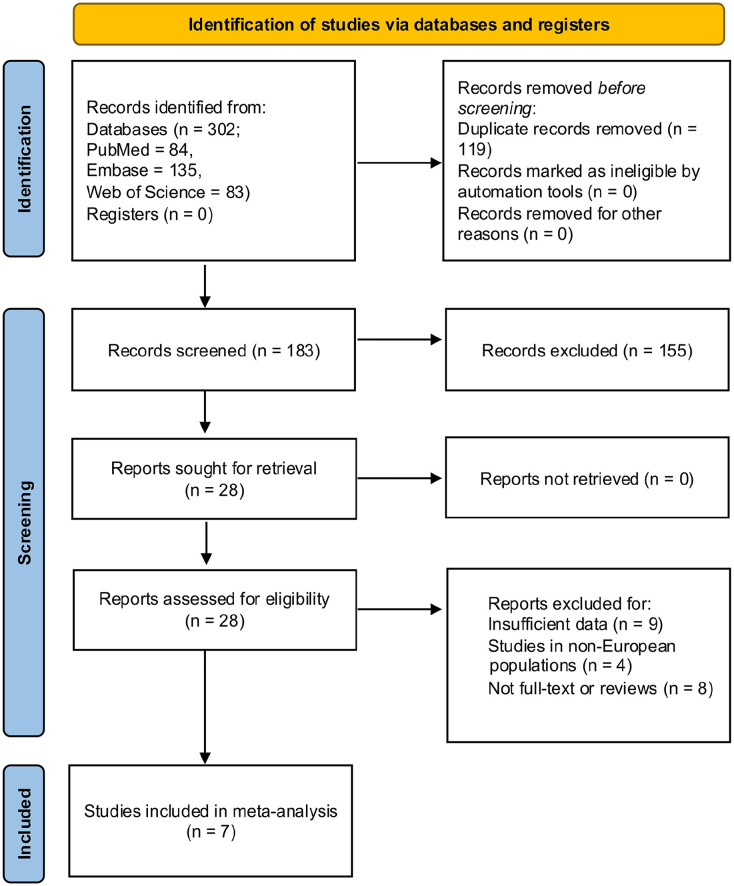
Flow diagram of study selection process for meta-analysis. A total of 302 studies were identified using online database search and 7 studies were finally selected for meta-analysis. The flow diagram was prepared in accordance with PRISMA 2020 guidelines.^23^

A systematic search of the PubMed, Embase, and Web of Science (WoS) databases, from the date of inception to September 2024, was carried out to identify all published articles that investigated the circulating (plasma/serum) levels of adiponectin in SSc patients (Figure 2). For this purpose, the following key words or search terms were used: “systemic sclerosis” and/or “scleroderma” combined with “adiponectin” and/or “adipokines.” All references cited in the above articles were also searched on three databases and reviewed to identify any additional studies of interest. No restrictions were applied for language, country or race. For final recruitment, only the data from full-text-published articles were obtained. Two authors (T.F. and C.M.) independently conducted the systematic literature search, data extraction, and check for the studies to be included in the meta-analysis.

### Inclusion and exclusion criteria

The following inclusion criteria were used for a study to be eligible for meta-analysis: (a) they were case-control studies for SSc patients and comparative healthy controls, (b) the study participants were humans, (c) studies provided detailed data for circulating (plasma/serum) levels of adiponectin, both in healthy controls and SSc patients. To be comparable to the following MR analysis and avoid repetition of previous meta-analysis studies, only studies in European (or Caucasian) individuals were considered for meta-analysis.

The studies were excluded from the meta-analysis if (a) they were reviews, case reports, or conference abstracts; (b) the detailed data on adiponectin levels in SSc patients and healthy controls were not accessible; (c) studies were not performed in humans; (d) more than 30% of the population included in the study were non-Europeans/Caucasians.

### Literature quality assessment

The Newcastle-Ottawa quality assessment scale (NOS)^24^ was used to assess the study quality. The NOS is based on eight questions that are categorized into three aspects to calculate a “study quality assessment score,” that is, selection of the study groups, comparability of the study groups and ascertainment of exposure (for case–control studies). The NOS represent scores from 0 to 9, with 9 being the highest possible score that, in turn, indicates a high methodological quality.

### Data extraction

After reviewing each selected article, the information for primary author’s name, year of publication, country of origin, ethnicity, study design, number of cases and controls, data for mean adiponectin levels, their standard deviation (SD), and *p*-values of the comparison test were extracted. In scenarios where the data given was median, quartile 1 to quartile 3, interquartile range or range, the mean and SD values were computed.^25,26^

### Statistical analysis

Meta-analysis was performed to calculate the standardized mean difference (SMD) and corresponding 95% confidence interval (CI) using the R package “metafor” (v4.6-0).^27^ The between-study heterogeneity was tested using Cochran’s *Q* test, with a *p*-value of < 0.05 as an indicative of significant heterogeneity. The effect of heterogeneity was quantified using the *I*^2^ = 100% × (*Q*-df)/*Q* equation.^28^ The *I*^2^ represents the proportion of variability that is explained by differences between the studies included in the meta-analysis rather than by chance. The values for *I*^2^ range between 0% and 100%, with a higher value indicating a higher heterogeneity. For meta-analysis, a *Q* test *p* < 0.05 and *I*^2^ < 50% was taken as a cut-off to fit either fixed effect (with values lower than cut-off) or random effect (with values higher than cut-off) models. The prospective publication bias was estimated by Begg’s rank test,^29^ Egger linear regression test,^30^ as well as by visually inspecting the funnel plot.

### MR analysis

The MR analysis in this study was conducted in accordance with the Strengthening the Reporting of Observational Studies in Epidemiology using Mendelian Randomization (STROBE-MR) guidelines.^31^

### Data sources

To assess the causal relationship between adiponectin and SSc, a two-sample, bidirectional, MR approach was applied. Specifically, we evaluated whether genetically predicted adiponectin levels influence SSc risk (forward MR), and conversely, whether a genetic liability to SSc influences circulating adiponectin levels (reverse MR). Summary-statistics for variant-adiponectin associations were obtained from ADIPOGen consortium,^32^ which represents the largest meta-analysis of genome-wide association studies (GWAS) for serum adiponectin levels in individuals of European ancestry (*n* > 39,000). The variant–SSc association summary-statistics data were sourced from López-Isac et al.^33^ The study is so far the largest (*n* > 26,000 participants) and most comprehensive SSc GWAS in European population. The details of the data extracted are provided in Tables S1 and S2 in Supplementary file.

### Instrument selection

We used a set of nine variants (single-nucleotide polymorphisms or SNPs) associated with serum adiponectin levels at a genome-wide significance (*p* < 5 × 10^-08^) in the ADIPOGen consortium as the adiponectin instrument (Supplementary file: Table S2). To assess the causal effect of genetic liability to SSc on adiponectin levels, we constructed an instrument using 21 variants associated with SSc risk, as well as three variants for the dcSSc subtype and 13 variants for the lcSSc subtype, all selected from López et al.^33^ at a genome-wide significance (*p* < 5 × 10^-08^) (Supplementary file: Table S2). We followed the three key assumptions of MR for relevance (IVs are strongly associated with the exposure), exchangeability (IVs are not associated with confounders), and exclusion restriction (IVs influence the outcome only through the exposure) while selecting the IVs. Sensitivity analyses (described below), including MR-Egger and heterogeneity tests, were conducted to check for potential violations of these assumptions.

For all variants included in the MR instrument, rigorous allele harmonization was performed between exposure and outcome datasets,^34^ which involved effect allele matching, consistency in directionality and exclusion of palindromic variants. For both serum adiponectin and SSc risk, independent variants were selected as instrumental variables (IVs) (linkage disequilibrium or LD: all *r*^2^ < 0.1). Proxy variants from Ensembl (Human: GRCh38.p14),^35^ at an LD *r*^2^ = 1, were used where the information for a variant was unavailable in the outcome dataset. Instrument strength was evaluated using *F*-statistics, calculated using the formula (*R*^2^/K)/[(1-*R*^2^) (N-K-1)), where “K” represents the number of variants and “*N*” is the sample size. *R*^2^ in this equation represents the total variance explained via IV, which is calculated using 2*EAF(1-EAF) β^2^, where EAF represents the effect allele frequency while β is the variant-adiponectin association estimate. An *F*-statistic >10 was considered indicative of adequate instrument strength.^36^

### Main MR analysis

For the main MR analysis, causal estimates for each variant were calculated using Wald ratio estimator.^37^ The estimator provides the ratio of the variant-outcome estimate over variant-exposure estimate. The standard error for the estimates was calculated using Delta method.^38^ These estimates were then pooled using random-effects inverse variance-weighted (IVW) method.^39^ The IVW method is applied assuming the absence of any pleiotropic effects of the variants used as IV on the outcome (SSc or adiponectin). To account for potential horizontal pleiotropy, three sensitivity MR analyses; simple median, weighted median, and MR-Egger, were applied. We also performed an outlier correction test, Mendelian Randomization Pleiotropy RESidual Sum and Outlier (MR-PRESSO), which is used to detect horizontal pleiotropy in multi-instrument MR.^40^ In addition, leave-one-out analysis was conducted to assess the influence of an individual variant on causal estimate. The details of these sensitivity analyses are provided in Supplementary file. All statistical analyses were performed using MendelianRandomization package (v0.9.0) in R (v4.4.1).^41^ A *p* < 0.05 was designated as statistically significant.

## Results

### Meta-analysis of the observational studies

In total, we have identified 302 studies through a systematic search of three electronic databases (84 from PubMed, 135 from Embase and 83 from WoS). After removing the duplicates and irrelevant studies following the set inclusion and exclusion criteria, seven studies were finally selected for the meta-analysis.^16,18,19,42–45^ Figure 2 illustrates the study identification, screening, and selection process in detail completed following the PRISMA 2020 guidelines.^23^ The selected studies included, in total, data from 439 SSc cases (dcSSc = 181, lcSSc = 258) and 274 healthy controls compared for serum or plasma adiponectin levels in European individuals from different regions. The NOS score was used to assess the study quality. For this study, the NOS score ranged from 6 to 8 (Table 1).

**Table 1.** Characteristic of the studies included in the meta-analysis for serum adiponectin levels in SSc patients.

No.	Source	Region	European % (case: control)	Case			Control			Criteria for SSc diagnosis	NOS
				*N*	Mean	SD	*N*	Mean	SD		
**dcSSc** **+** **lcSSc**											
1a	Lakota et al.^42^	Caucasian from USA	71:100	129	14.7	11.55	86	15.3	8.44	NA	6
2	Tomčík et al.^43,a^	Czech Republic	100:100	39	7.33	2.46	30	7.84	2.57	ACR	8
3	Olewicz-Gawlik et al.^44,a^	Poland	100:100	29	23.2	15.75	30	22.1	8.48	ACR/EULAR	7
4a	Michalska-Jakubus et al.^18,b^	Poland	100:100	48	11.87	8.87	38	10.8	5.63	ACR/EULAR	7
5a	Stochmal et al.^16^	Poland	100:100	100	5.15	4.71	20	8.84	4.34	ACR/EULAR	7
6a	Dopytalska et al.^19,c^	Poland	100:100	58	7.97	3.76	30	8.65	4.82	ACR/EULAR	8
**dcSSc**											
1b	Lakota et al.^42^	Caucasian from USA	68:100	50	10.1	8.63	86	15.3	8.44	NA	6
7	Winsz-Szczotka et al.^45^	Poland	100:100	36	5.12	2.32	40	16.8	10.1	ACR	7
4b	Michalska-Jakubus et al.^18,b^	Poland	100:100	6	8.63	10.38	38	10.8	5.63	ACR/EULAR	7
5b	Stochmal et al.^16^	Poland	100:100	26	4.52	4.39	20	8.84	4.34	ACR/EULAR	7
6b	Dopytalska et al.^19,c^	Poland	100:100	40	7.83	4.14	30	8.65	4.82	ACR/EULAR	8
**lcSSc**											
1c	Lakota et al.^42^	Caucasian from USA	75:100	79	18.3	9.89	86	15.3	8.44	NA	6
4c	Michalska-Jakubus et al.^18,b^	Poland	100:100	42	12.42	8.61	38	10.8	5.63	ACR/EULAR	7
5c	Stochmal et al.^16^	Poland	100:100	74	5.24	4.83	20	8.84	4.34	ACR/EULAR	7
6c	Dopytalska et al.^19,c^	Poland	100:100	18	8.14	3.15	30	8.65	4.82	ACR/EULAR	8

*N*: number of participants in each group, Mean: mean value of serum/plasma adiponectin levels in µg mL^−1^, SD: standard deviation of mean, SSc: Systemic sclerosis, dcSSc: diffuse cutaneous SSc, lcSSc: limited cutaneous SSc, USA: United States of America, EULAR: European League Against Rheumatism, ACR: American College of Rheumatology, NOS: Newcastle-Ottawa quality assessment scale score.

a dcSS/lcSS = *N* = 12/27 in Tomcik et al. 2012, and 11/18 in Olewicz-Gawlik et al. 2015; both studies did not provide information regarding serum/plasma adiponectin levels stratified for SSc (dcSSc and lcSSc) subgroups that brought the total count of dcSSc and lcSSc cases to 158 and 213, respectively, to be included in the subgroup meta-analysis.

b The study included only female participants.

c The study involved a follow-up of 6 and 9 months for SSc patients.

**Figure 3.**
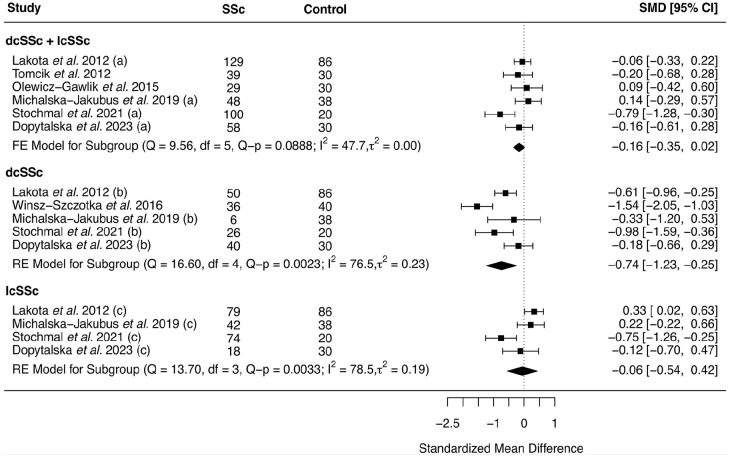
Forest plot for meta-analysis of the published data from observational case–control studies comparing serum adiponectin levels between systemic sclerosis (SSc) patients and healthy controls in overall and stratified by SSc subgroups; results are presented as standardized mean difference (SMD) and its corresponding 95% confidence interval (CI). The labels “a,” “b,” and “c” in front of the study name indicates data extracted, respectively, for dcSSc + lcSSc, dcSSc and lcSSc subgroups, from the same study. lcSSc: limited cutaneous systemic sclerosis, dcSSc: diffuse cutaneous systemic sclerosis, FE: fixed effects model, RE: random effects model.

The test for heterogeneity was significant in dcSSc (*Q-p* = 0.002) and lcSSc (*Q-p* = 0.003) subgroups but not dcSSc + lcSSc combined group (*Q-p* = 0.08) (Figure 3, Supplementary file: Table S1). The fixed-effects meta-analysis in SSc combined group was suggestive for lower serum adiponectin levels in SSc patients than healthy controls (SMD (95% CI) = −0.16 (−0.35 to 0.02)), albeit not statistically significant (*p* = 0.07). The subgroup-stratified meta-analysis, using random effects model, revealed significantly lower adiponectin levels in dcSSc patients (SMD (95% CI) = −0.74 (−1.23 to −0.25), *p* = 0.003) compared to controls, while no significant difference was observed in lcSSc patients (SMD (95% CI) = −0.06 (−0.54 to 0.42), *p* = 0.81) (Figure 3, Supplementary file: Table S3). No significant publication bias was found for the meta-analysis with no change in the symmetry of the funnel plot, both via Egger’s regression (*t* = −0.62, *p* = 0.56) and Begg’s rank (Kendall’s tau = −0.33, *p* = 0.46) tests. The results of dcSSc and lcSSc subgroups are detailed in Supplementary file: Table S3.

### Forward MR analysis

**Figure 4.**
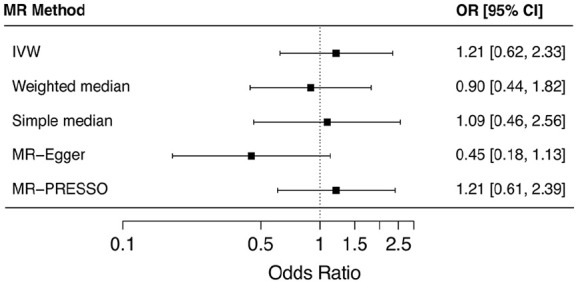
Forest plot illustrating the results of two-sample, forward, Mendelian randomization (MR) analysis for a causal effect of circulating adiponectin levels on SSc risk. Causal estimates from a set of nine single nucleotide variants (or SNPs) are summarized as inverse variance-weighted (IVW) estimates while simple median, weighted median, MR-Egger and MR-PRESSO estimates are presented as sensitivity analyses. Results are presented as odds ratios (ORs) and their corresponding 95% CI.

To determine if genetically predicted adiponectin levels have a causal effect on SSc risk, we have performed a forward MR analysis. Table S4 and Figure S1 in Supplementary file provides the causal estimates for single-SNP analysis in forward MR. All odds ratios (ORs) in forward MR analyses are presented for a change in SSc risk per 1 µg mL^−1^ increase in serum adiponectin levels. The main MR analysis, using IVW method, did not provide evidence of a causal effect of adiponectin levels on the risk of SSc in Europeans (OR (95% CI) = 1.21 (0.62 to 2.33), *p* = 0.57) (Figure 4). There was no significant heterogeneity across the estimates of variants included in adiponectin instrument (Cochran’s *Q* = 12.7, *p* = 0.12) (Supplementary file: Figure S1). Applying the sensitivity analyses to account for horizontal pleiotropy also did not indicate any adiponectin to SSc risk causal effect (weighted median: OR (95% CI) = 0.89 (0.44 to 1.82), *p* = 0.76; simple median: OR (95% CI) = 1.09 (0.46 to 2.56), *p* = 0.84; MR-Egger: OR (95% CI) = 0.45 (0.18 to 1.13), *p* = 0.08, *p*_intercept_ = 0.01) (Figure 3). The outlier detection test MR-PRESSO did not identify any outlier variant (OR (95% CI) = 1.21 (0.61 to 2.39), *p*_raw_ = 0.59, *p*_global_ = 0.14). In addition, leave-one-out sensitivity analysis did not indicate any single variant to be significantly affecting the causal estimates in the IVW MR analysis (Supplementary file: Figure S2).

### Reverse MR analysis

**Figure 5.**
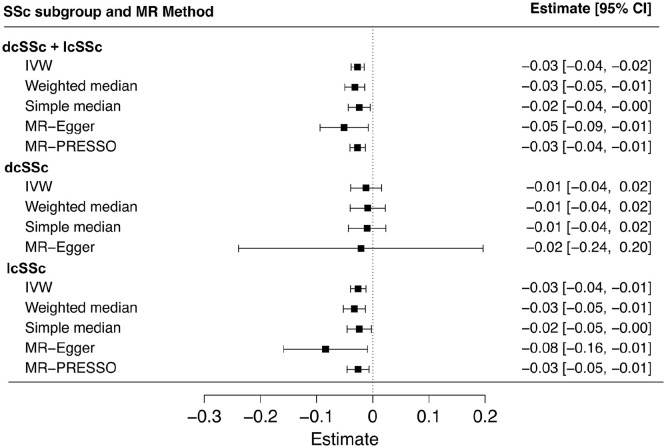
Forest plot illustrating the results of two-sample, reverse, Mendelian randomization (MR) analysis for a causal effect of genetic liability to SSc on circulating adiponectin levels. Causal estimates from a set of 21 (dcSSc + lcSSc), 3 (dcSSc), and 13 (lcSSc) variants or single nucleotide variants (or SNPs) are summarized as inverse variance-weighted (IVW) estimates, while simple median, weighted median, MR-Egger, and MR-PRESSO estimates are presented as sensitivity analyses. Results are presented as regression estimates (ß) and their corresponding 95% CI. Owing to the limited number of variants in the dcSSc instrument, MR-PRESSO analysis was not feasible for this subgroup.

In the reverse direction, we investigated whether genetic liability to SSc causally affects circulating adiponectin levels. All estimates (or ß) in reverse MR analyses are presented for a change in serum adiponectin levels in µg mL^−1^ per unit increase in log odds of SSc. Tables S5 and Figure S3 in Supplementary file provides the causal estimates for single-SNP analysis in reverse MR. The main MR analysis, using the IVW method, provided evidence for a causal association between a genetic liability to SSc and lower circulating adiponectin levels in the overall SSc group (dcSSc and lcSSc combined) (ß (95% CI) = −0.027 (−0.04 to −0.02), *p* = 6.8E-06) (Figure 5). In subgroup analyses, a significant causal association was observed specifically in the lcSSc group (ß (95% CI) = −0.026 (−0.04 to −0.01), *p* = 0.002) (Figure 5), while no causal effect was detected in the dcSSc group (ß (95% CI) = −0.012 (−0.04 to 0.02), *p* = 0.39) (Figure 5). No evidence of significant heterogeneity was found in the IVW analysis for the combined group (Cochran’s *Q* = 20.6, *p* = 0.41) (Supplementary file: Figure S3). Sensitivity analyses supported the findings in the overall group (weighted median ß (95% CI) = −0.032 (−0.05 to −0.01), *p* = 3.7E-04; simple median: ß (95% CI) = −0.024 (−0.04 to −0.005), *p* = 0.01; MR-Egger: ß (95% CI) = −0.051 (−0.09 to −0.01), *p* = 0.02, *p*_intercept_ = 0.25). The results for test of heterogeneity and sensitivity analyses for the dcSSc and lcSSc subgroups are detailed in Figure 5. MR-PRESSO did not indicate an outlier variant for overall (ß (95% CI) = −0.027 (−0.04 to −0.01), *p*_raw_ = 3.1E-04, *p*_global_ = 0.41) and lcSSc subgroup (ß (95% CI) = −0.025 (−0.05 to −0.01), *p*_raw_ = 0.01, *p*_global_ = 0.21), while it was not possible to conduct MR-PRESSO for dcSSc group owing to the limited number of IV variants (Figure 5). Leave-one-out analysis confirmed that no single variant significantly influenced the causal estimates in the overall or subgroup analyses (Supplementary file: Figure S4).

## Discussion

In this study, we conducted an updated meta-analysis and a range of bidirectional (forward and reverse), two-sample MR analyses to systematically explore whether adiponectin levels are affected in SSc and whether there exists a causal relationship between genetically predicted adiponectin levels and the development of SSc. Our meta-analysis of observational studies was only suggestive of lower levels of circulating adiponectin in SSc patients than controls, when dcSSc and lcSSc subgroups were analyzed together. However, there was a significant decrease in adiponectin levels in patients with dcSSc, but not lcSSc, compared to healthy controls. Moreover, our forward MR analysis did not support a causal effect of genetically predicted circulating adiponectin levels on the risk of developing SSc. In contrast, reverse MR analysis revealed that genetic liability to SSc is causally associated with lower levels of circulating adiponectin.

Adiponectin is a cytokine mostly produced by the adipose tissue, and hence, is a so-called adipokine. Circulating adiponectin levels are not dependent on the mass of the adipose tissue but on the functionality of the adipocytes. In case of adipocyte dysfunction, as for obesity, circulating adiponectin levels are markedly decreased.^46^ Dysfunction of the dermal white adipose tissue occurs in SSc, supporting the hypothesis that this leads to low circulating adiponectin levels as seen in SSc.^13,47^ Previous studies have shown lower circulating levels of adiponectin in patients with SSc compared to controls.^45,48^ Nevertheless, the small sample sizes in these studies imply a lack of statistical power to support or refute the hypothesis that adiponectin levels are altered in SSc. Here, we pooled the data of seven observational studies in a meta-analysis that overall included 439 SSc patients and 274 controls of European descendance. Our meta-analysis showed that circulating adiponectin levels were lower in patients with SSc compared to controls, although the association was only suggestive since it lacked statistical significance. However, in the meta-analysis stratified for SSc subgroups, we found significantly lower levels of circulating adiponectin only in patients with dcSSc, but not in those with lcSSc compared to controls. This finding supports the hypothesis that dermal adipose tissue dysfunction in SSc contributes to the reduced levels of circulating adiponectin, as patients with dcSSc exhibit more extensive skin involvement than those with lcSSc.

To date, two meta-analyses have investigated the relationship between circulating adipokines and SSc.^14,15^ While the published data searched in each of these meta-analyses was up to the beginning of 2016, our study updated the literature search through September 2024, incorporating the data from three additional recent case–control studies,^16,18,19^ which in turn enhanced the statistical power of our meta-analysis. Earlier meta-analyses reported that SSc patients had lower circulating adiponectin levels compared to healthy controls across both European and Asian populations. These studies also reported outcomes stratified by populations, with results in European individuals aligning with our updated meta-analysis. While the direction of association is comparable among all subgroups, the significant heterogeneity in subgroup-stratified meta-analysis in our study is reflective of inconsistent findings reported in individuals from different European countries. Previous studies have also reported other factors like age,^14,43^ disease duration, type and activity,^14,15,44^ and even study design (e.g. methodological variability or different assay techniques) to be possible contributors to such heterogeneity. Moreover, factors like smaller sample size (hence lack of statistical power to detect an effect), both population (genetic backgrounds within Europe, environmental exposures, and lifestyle factors) and clinical (organ involvement or treatment history) heterogeneities as well as confounding variables could also be possible contributors to inconsistent and variable association across different European populations. This not only suggests the need for a cautious interpretation of the meta-analysis results but also warrants similar studies with larger sample size.

Although the exact relationship between adiponectin levels and SSc remains unexplained, preceding reports in literature suggest that reduced adiponectin in SSc may play a pathogenic role, given its potential anti-fibrotic effects on skin fibroblasts.^13^ This effect is supposedly mediated via adenosine monophosphate protein-activated kinase activation.^49^ In an animal model, an adiponectin agonist drug improved bleomycin-induced dermal fibrosis in mice.^50^ Our findings of decreased adiponectin levels in SSc patients, particularly those with diffuse cutaneous involvement (dcSSc), aligns with the proposed anti-fibrotic and immunomodulatory properties of adiponectin. Adiponectin is known to interfere with the TGF-β/SMAD signaling cascade, thereby inhibiting collagen production and fibroblast activation—two hallmarks of SSc pathophysiology.^11,49^ Moreover, adiponectin modulates immune function by suppressing the secretion of IL-6 and TNF-α and inhibiting macrophage activation,^12,51^ which are relevant to the chronic inflammatory milieu observed in SSc. These findings suggest that reduced adiponectin levels in SSc may represent a loss of a crucial protective signal rather than a secondary effect.

Building on these insights, we investigated the causal association and its directionality between adiponectin and SSc risk by performing a series of complementary bidirectional MR analyses using publicly available GWAS summary data. Given the clinical findings where adiponectin levels negatively correlated with disease duration and skin thickness in SSc,^19,52^ reverse causality was a plausible hypothesis, hence, the reverse MR analysis was also conducted. This MR study did not find a causal relationship between adiponectin levels and the risk of developing SSc in forward MR. However, we report a genetic liability to SSc to be causally associated with lower levels of circulating adiponectin. Although the direction of the causal effect was consistent across multiple robust MR analyses, the association remained statistically significant only in the combined (lcSSc and dcSSc) group and the lcSSc-only subgroup, but not in the dcSSc-only subgroup. We believe this may be partly due to the availability of limited genetic variant data specifically associated with dcSSc. For example, only three variants were available in this study to construct an MR instrument for dcSSc, which might have contributed to the observed findings. This highlights the need for larger and more comprehensive GWA studies focused on SSc with particular emphasis on its subtypes, to improve the power of genetic instruments and enhance the accuracy of causal inference in future MR analyses. Taken together, the findings from our MR analyses suggest that it is indeed a genetic liability to SSc that causes lower circulating adiponectin levels, rather than adiponectin levels influencing the risk of SSc. While there requires more explanation for this causality, our results are in line with above clinical observations showing disease duration and severity coinciding with lower adiponectin levels.

Our study is not without limitations. First, all the studies included in the meta-analysis had rather small number of participants. This unfortunately is an inherent limitation in the field of SSc due to the rarity of the disease. Second, various unidentified or confounding variables may have contributed to the results in our meta-analysis as the pooled data were obtained from purely observational studies. Further studies in larger samples with more comprehensive clinical data are desirable. Third, we have limited both analyses included in this study to individuals of European descendent. Our results, therefore, are not generalizable on populations with other ancestral background. Future efforts should focus on including non-European populations to enhance cross-ethnic applicability of our findings.

The strengths of our study go hand in hand with above-described limitations. We have here presented an updated meta-analysis (since 2017) to assess the change in circulating adiponectin levels in SSc, including the largest pooled data in European individuals to date that provided additional statistical power to the analysis. We have performed the first ever MR analysis to address confounding and to elucidate any causal relationship between circulating levels of adiponectin and the risk of developing SSc. Moreover, to make the MR outcomes more robust, we have used a series of complementary MR methods.

## Conclusion

Our meta-analysis of observational, case–control studies in European populations supports the finding of lower circulating levels of adiponectin in patients with SSc compared to controls, with this reduction being more pronounced in the dcSSc subtype than in lcSSc, although both subtypes showed a similar association trend. Our MR analysis did not indicate a causal effect of genetically predicted adiponectin levels on SSc risk; however, it does provide evidence that a genetic liability to SSc causally contributes to lower circulating adiponectin levels. Nevertheless, further studies in larger cohorts, possibly with more enriched data on SSc-related genetic variants, and in populations of different ancestries are warranted to help determine whether our findings are generalizable across different ethnic groups and enhance our understanding of the disease’s genetic and environmental influences.

## Supplemental Material

sj-pdf-1-jso-10.1177_23971983251352341 – Supplemental material for Circulating adiponectin levels in systemic sclerosis: A meta-analysis and bidirectional Mendelian randomization study Supplemental material, sj-pdf-1-jso-10.1177_23971983251352341 for Circulating adiponectin levels in systemic sclerosis: A meta-analysis and bidirectional Mendelian randomization study by Tahzeeb Fatima, Cecilia Överdahl and Cristina Maglio in Journal of Scleroderma and Related Disorders
