# Supplementary material for: Circulating adiponectin levels in systemic sclerosis: A meta-analysis and bidirectional Mendelian randomization study
Source: J Scleroderma Relat Disord. 2025 Jul 17;10(3):483–93. doi: 10.1177/23971983251352341 (PMC12271137; doi:10.1177/23971983251352341)
Supplement: sj-pdf-1-jso-10.1177_23971983251352341 – Supplemental material for Circulating adiponectin levels in systemic sclerosis: A meta-analysis and bidirectional Mendelian randomization study [file sj-pdf-1-jso-10.1177_23971983251352341.pdf]

Sensitivity MR analyses

Weighted median [1] and MR-Egger regression [2] were used as main sensitivity analyses in the two-sample MR performed in our study. These analyses have less stringent assumptions on horizontal pleiotropic effect of genetic instruments on the considered outcome and are less vulnerable to bias if the MR assumptions does not hold [3]. The weighted median estimator provides the weighted median effect of all MR estimates produced by individual instruments, with weights equal to the inverse of the standard error. The weighted median estimate is valid when more than half of the variants included in the instruments are valid [1]. Additionally, the slope and intercept of the MR-Egger regression was estimated, which provides average horizontal pleiotropy, and an MR-estimate adjusted for pleiotropy, respectively. An MR-Egger intercept value of $p < 0.05$ was considered as indicative of pleiotropy [2]. To test the robustness of our analysis to the choice of variants included in the adiponectin instrument, we performed leave-one-out analysis. This analysis provides the MR estimates by removing one variant from the IVW analysis in each turn. This analysis assesses if any single variant is driving the causal effect of exposure on the outcome considerably.

Table S1 Genome-wide significant variants of adiponectin levels and their association with Systemic sclerosis (SSc)

Gene/closest gene	SNP	Chr	EA	EAF	Adiponectin			Systemic sclerosis			
					β	SE	p-value	Proxy SNP	β	SE	p-value
LYPLAL1	*rs3001032*	1	T	0.7	-0.02	0.004	3.6E-08	No	-0.015	0.022	0.507
GNL3	*rs1108842*	3	C	0.5	0.03	0.004	1.39E-13	No	0.036	0.023	0.125
ADIPOQ	*rs6810075*	3	T	0.6	0.06	0.004	1.8E-43	No	0.005	0.041	0.899
ADIPOQ	*rs16861209**	3	A	0.08	0.16	0.009	2.7E-64	*rs17300539*	-0.076	0.076	0.313
PDE3A	*rs7955516**	12	C	0.4	0.02	0.004	4.45E-08	*rs7303397*	0.051	0.028	0.072
GPR109A	*rs601339*	12	G	0.2	0.03	0.005	7.81E-10	No	0.066	0.03	0.026
CMIP	*rs2925979*	16	T	0.3	-0.04	0.005	1.21E-20	No	0.029	0.034	0.386
CDH13	*rs12922394*	16	T	0.1	-0.08	0.01	1.99E-15	No	0.009	0.058	0.876
PEPD	*rs731839**	19	G	0.35	-0.03	0.004	7.97E-12	*rs7256564*	0.037	0.055	0.499

SNP: Single nucleotide polymorphism, Chr: Chromosome, EA: Effect allele, OA: Other allele, EAF: Effect allele frequency.

*Proxy SNPs were used at an LD $r^2 = 1$ as the original SNP was not available in the outcome GWAS (Lopez et al.) data. Proxies were obtained from Ensembl (Human: GRCh38.p14) [4]

Table S2 Genome-wide significant variants for Systemic sclerosis (SSc) and their association with adiponectin levels

Gene/closest gene	SNP	Chr	EA	MAF	Systemic sclerosis			Adiponectin			
					β	SE	p-value	Proxy SNP	β	SE	p-value
**SNPs for dcSSc and lcSSc combined**											
IL12RB2	*rs3790566*	1	T	0.24	0.148	0.024	3.84E-10	No	-0.004	0.005	0.498
CD247	*rs2056626*	1	G	0.39	-0.211	0.031	1.31E-11	No	-0.012	0.005	0.013
TNFSF4- LOC100506023 - PRDX6	*rs2022449*	1	T	0.23	0.14	0.026	6.28E-08	No	-0.003	0.005	0.57
TNFSF4- LOC100506023 - PRDX6	*rs1857066*	1	A	0.25	-0.139	0.024	5.02E-09	No	-0.004	0.005	0.394
NAB1	*rs16832798*	2	C	0.14	0.166	0.028	5.2E-09	No	0.006	0.006	0.408
STAT4	*rs3821236*	2	A	0.2	0.27	0.027	1.94E-23	No	0.011	0.005	0.045
STAT4	*rs4853458**	2	A	0.23	0.3	0.035	4.86E-18	*rs11889341*	-0.014	0.005	0.01
FLNB -DNASE1L3-PXK	*rs7355798**	3	T	0.24	0.131	0.023	1.24E-08	*rs9884098*	0.004	0.005	0.467
FLNB-DNASE1L3- PXK	*rs4076852*	3	G	0.26	0.148	0.023	1.04E-10	No	-0.003	0.005	0.523
POGLUT1-TIMMDC1-CD80- ARHGAP31	*rs9884090*	3	A	0.16	-0.186	0.029	1.89E-10	No	0.001	0.006	0.884
IL12A	*rs589446**	3	T	0.35	-0.151	0.024	1.95E-10	*rs485789*	0.011	0.004	0.016
DGKQ	*rs11724804*	4	A	0.44	0.157	0.024	5.31E-11	No	-0.002	0.005	0.689
NFKB1	*rs230534*	4	T	0.34	0.14	0.024	5.38E-09	No	0.006	0.005	0.222
TNIP1	*rs3792783*	5	G	0.16	0.182	0.026	2.42E-12	No	-0.002	0.006	0.794
ATG5	*rs633724*	6	T	0.35	0.122	0.021	2.84E-09	No	0.004	0.004	0.436
IRF5- TNPO3	*rs36073657**	7	T	0.1	0.336	0.036	3.1E-21	*rs12539476*	0.003	0.007	0.69
FAM167A-BLK	*rs2736340*	8	T	0.24	0.215	0.023	3.33E-21	No	-0.001	0.005	0.824
TSPAN32,CD81-AS1	*rs2651804*	11	T	0.17	-0.198	0.031	2.54E-10	No	0.006	0.006	0.276
DDX6	*rs11217020*	11	A	0.2	-0.174	0.026	2.08E-11	No	-0.002	0.005	0.769
CSK	*rs1378942*	15	C	0.39	0.166	0.022	1.84E-14	No	-0.012	0.004	0.01

IL12RB1	*rs2305743*	19	A	0.2	-0.186	0.03	4.64E-10	No	-0.007	0.006	0.209
**SNPs for dcSSc**											
NAB1-STAT4	*rs716254**	2	A	0.15	0.262	0.045	4.369E-09	*rs16832798*	0.006	0.006	0.408
DDX6	*rs11217019**	11	A	0.2	-0.261	0.044	2.148E-09	*rs11217020*	-0.002	0.005	0.769
ANKRD12	*rs4798783**	18	G	0.26	0.199	0.036	3.967E-08	*rs8092613*	-0.002	0.006	0.744
**SNPs for lcSSc**											
CD247	*rs2056626*	1	G	0.39	-0.223	0.037	1.47E-09	No	-0.012	0.005	0.013
TNFSF4-LOC100506023-PRDX6	*rs10737299*	1	T	0.34	-0.159	0.027	2.69E-09	No	0.004	0.005	0.429
STAT4	*rs3821236*	2	A	0.2	0.289	0.032	7.77E-20	No	0.011	0.005	0.045
MERTK	*rs3761700*	2	G	0.46	0.144	0.025	1.04E-08	No	-0.002	0.004	0.671
FLNB-DNASE1L3-PXK	*rs7652027**	3	G	0.26	0.184	0.026	1.03E-12	*rs2176082*	0.003	0.005	0.533
IL12A	*rs485499*	3	C	0.35	-0.191	0.032	1.86E-09	No	-0.011	0.004	0.015
DGKQ	*rs11724804*	4	A	0.44	0.153	0.028	3.16E-08	No	-0.002	0.005	0.689
TNIP1	*rs3792783*	5	G	0.16	0.187	0.031	1.58E-09	No	-0.002	0.006	0.794
ATG5	*rs633724*	6	T	0.35	0.137	0.024	2.01E-08	No	0.004	0.004	0.436
FAM167A-BLK	*rs2736340*	8	T	0.24	0.216	0.027	9.33E-16	No	-0.001	0.005	0.824
TSPAN32, CD81-AS1	*rs2651804*	11	T	0.17	-0.224	0.038	3.70E-09	No	0.006	0.006	0.276
CSK	*rs1378942*	15	C	0.39	0.156	0.025	5.25E-10	No	-0.012	0.004	0.01
IL12RB1	*rs2305743*	19	A	0.2	-0.201	0.036	1.65E-08	No	-0.007	0.006	0.209

SNP: Single nucleotide polymorphism, Chr: Chromosome, EA: Effect allele, OA: Other allele, MAF: Minor allele frequency.

*Proxy SNPs were used at an LD $r^2 = 1$ as the original SNP was not available in the outcome GWAS (Dastani et al. 2012) data. For one of the dcSSc variants (*rs4798783*), the proxy variant was selected at an LD $r^2 = 0.84$ because of the unavailability of a proxy at $r^2 = 1$. Proxies were obtained from Ensembl (Human: GRCh38.p14) [4]

Table S3 Meta-analysis of circulating adiponectin levels in patients with SSc compared to controls

**SSc group/subgroup**	**No. of participants**		**Test of association**			**Test of heterogeneity**			**Egger's test**		**Begg's test**	
	**Case**	**Control**	**SMD**	**95% CI**	**p-value**	I^2 **(%)**	***Q***	***Q-p*-value**	**t**	***p*-value**	**Kendall's tau**	***p*-value**
**dcSSc + lcSSc**	403	234	-0.164	-0.345; 0.016	0.074	47.7	9.56	0.088	-0.62	0.565	-0.333	0.469
**dcSSc**	158	214	-0.741	-1.228; -0.253	0.003	76.5	16.6	0.002	-0.16	0.881	0.000	1.00
**lcSSc**	213	174	-0.057	-0.537; 0.423	0.815	78.5	13.7	0.003	-1.43	0.288	-0.666	0.333

SSc: Systemic sclerosis, dcSSc: diffuse cutaneous systemic sclerosis, lcSSc: limited cutaneous systemic sclerosis. SMD: standardized mean difference, *Q*: value of Chochran's Q test.

Table S4 Single SNP analysis for causal effect of adiponectin on Systemic sclerosis (SSc) risk

SNP	OR [95% CI]	p-value
rs3001032	2.12 [0.25 ; 18.28]	0.50
rs1108842	3.32 [0.74 ; 14.92]	0.12
rs6810075	1.09 [0.28 ; 4.15]	0.90
rs16861209	0.62 [0.25 ; 1.58]	0.32
rs7955516	12.81 [0.82 ; 199.14]	0.07
rs601339	9.03 [1.27 ; 64.07]	0.03
rs2925979	0.48 [0.09 ; 2.56]	0.39
rs12922394	0.89 [0.22 ; 3.70]	0.88
rs731839	0.29 [0.01 ; 10.59]	0.50

SNP: Single nucleotide polymorphism, OR: odds ratio, 95% CI (95% confidence interval)

Table S5 Single SNP analysis for causal effect of Systemic sclerosis (SSc) on adiponectin levels

SNP	OR [95% CI]	p-value
	**dcSSc + lcSSc**	
rs3790566	0.98 [0.91 ; 1.04]	0.49
rs2056626	0.95 [0.91 ; 0.99]	0.01
rs2022449	0.98 [0.91 ; 1.05]	0.56
rs1857066	0.97 [0.90 ; 1.04]	0.38
rs16832798	0.97 [0.90 ; 1.04]	0.39
rs3821236	0.96 [0.92 ; 1.00]	0.04
rs4853458	0.95 [0.92 ; 0.99]	0.01
rs7355798	0.97 [0.90 ; 1.05]	0.45
rs4076852	0.98 [0.92 ; 1.04]	0.51
rs9884090	1.00 [0.95 ; 1.07]	0.88
rs589446	0.93 [0.88 ; 0.98]	0.01
rs11724804	1.01 [0.95 ; 1.07]	0.68
rs230534	1.04 [0.98 ; 1.11]	0.21
rs3792783	0.99 [0.93 ; 1.06]	0.79
rs633724	1.03 [0.96 ; 1.11]	0.42
rs36073657	0.99 [0.95 ; 1.03]	0.68
rs2736340	0.99 [0.95 ; 1.04]	0.82
rs2651804	0.97 [0.91 ; 1.02]	0.26
rs11217020	0.99 [0.93 ; 1.05]	0.76
rs1378942	0.93 [0.88 ; 0.98]	0.01
rs2305743	0.96 [0.91 ; 1.02]	0.20
	**dcSSc**	
rs716254	0.98 [0.93 ; 1.03]	0.39
rs11217019	0.99 [0.95 ; 1.03]	0.76
rs4798783	0.99 [0.94 ; 1.05]	0.74
	**lcSSc**	
rs3821236	0.96 [0.93 ; 1.00]	0.04
rs2736340	0.99 [0.95 ; 1.04]	0.82
rs7652027	1.02 [0.97 ; 1.07]	0.52
rs1378942	0.93 [0.88 ; 0.98]	0.01
rs2056626	0.95 [0.91 ; 0.99]	0.01
rs3792783	0.99 [0.93 ; 1.06]	0.79
rs485499	0.94 [0.90 ; 0.99]	0.01
rs10737299	0.98 [0.92 ; 1.03]	0.42
rs2651804	0.97 [0.92 ; 1.02]	0.26
rs3761700	1.01 [0.96 ; 1.07]	0.66
rs2305743	0.96 [0.91 ; 1.02]	0.20
rs633724	1.03 [0.96 ; 1.09]	0.42
rs11724804	1.01 [0.95 ; 1.08]	0.68

SNP: Single nucleotide polymorphism, OR: odds ratio, 95% CI (95% confidence interval)

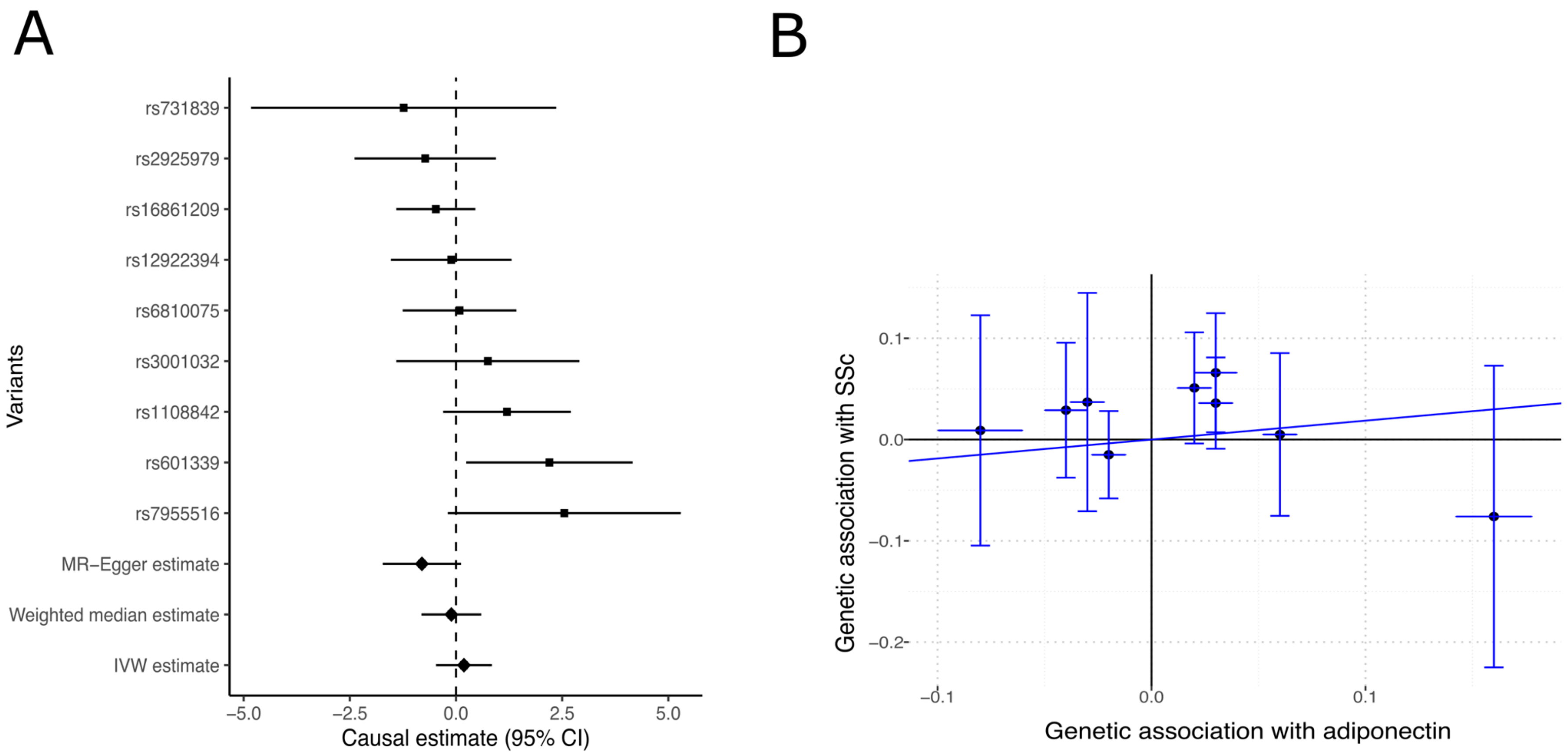

Figure S1 Results from forward MR analysis for causal effect of genetically predicted circulating adiponectin levels on Systemic sclerosis (SSc) risk; A) Single variant/SNP MR analysis, B) Effects of single variants on adiponectin and SSc

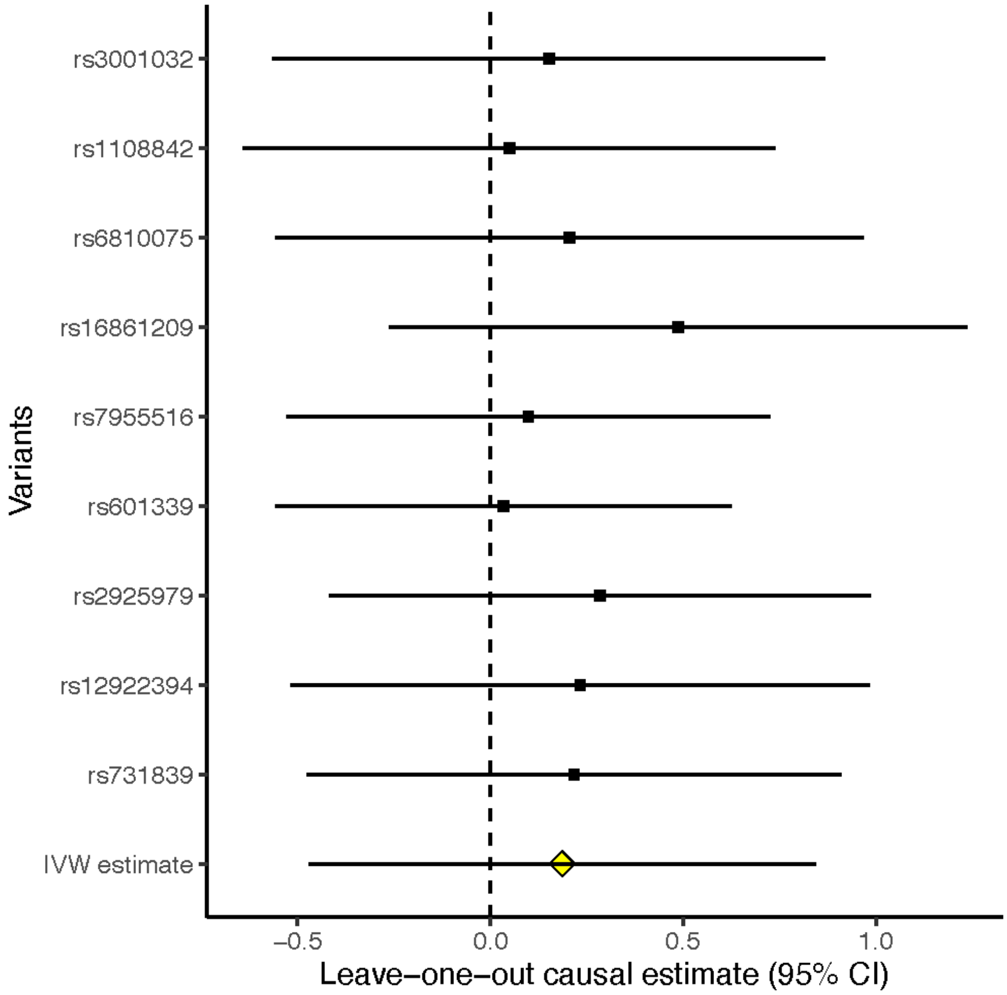

Figure S2 Leave-one-out sensitivity analysis for forward MR (adiponectin to SSc)

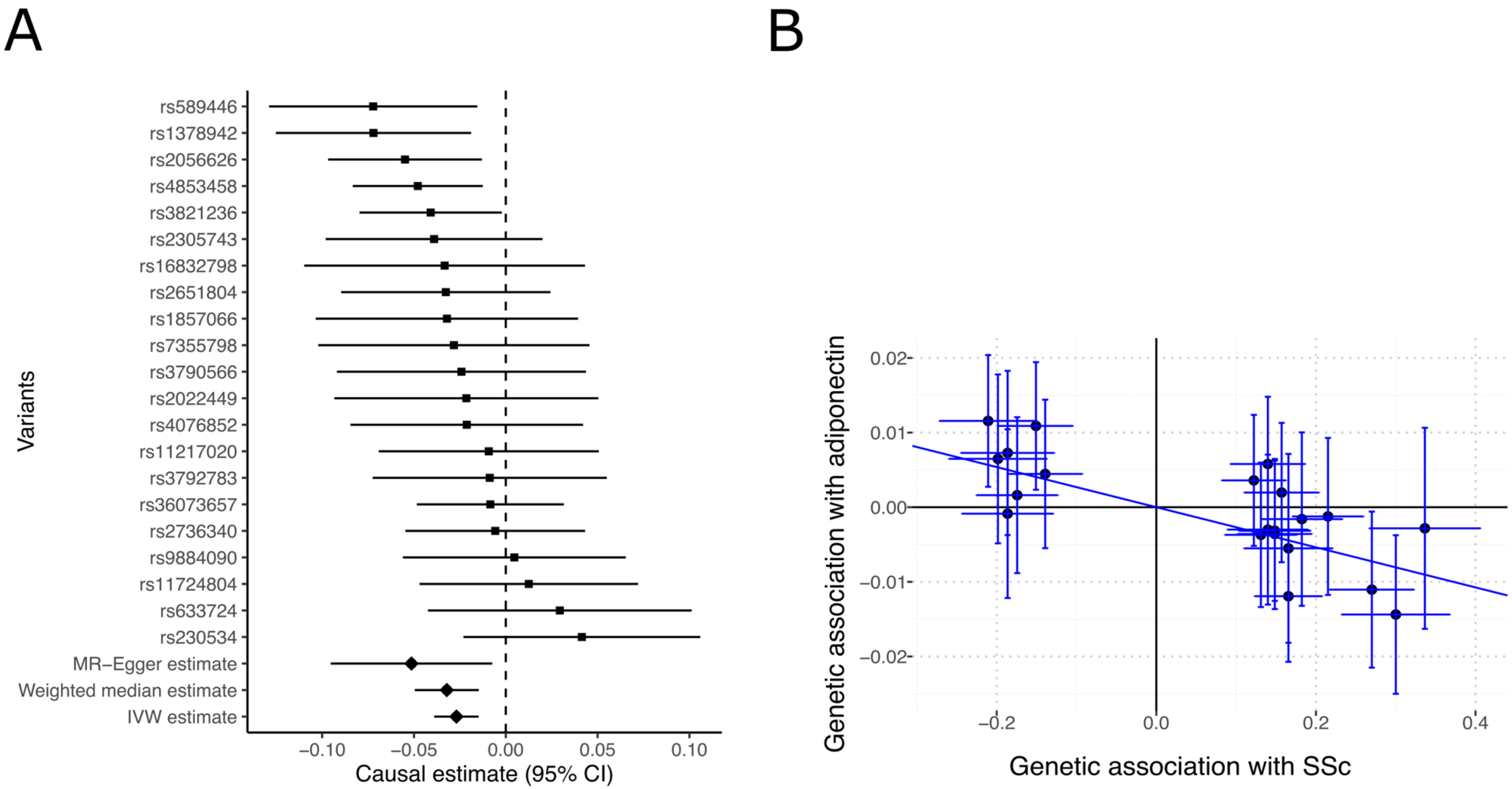

Figure S3 Results from reverse MR analysis for causal effect of genetic liability to Systemic sclerosis (SSc) on circulating adiponectin levels; A) Single variant/SNP MR analysis, B) Effects of single variants on SSc and adiponectin

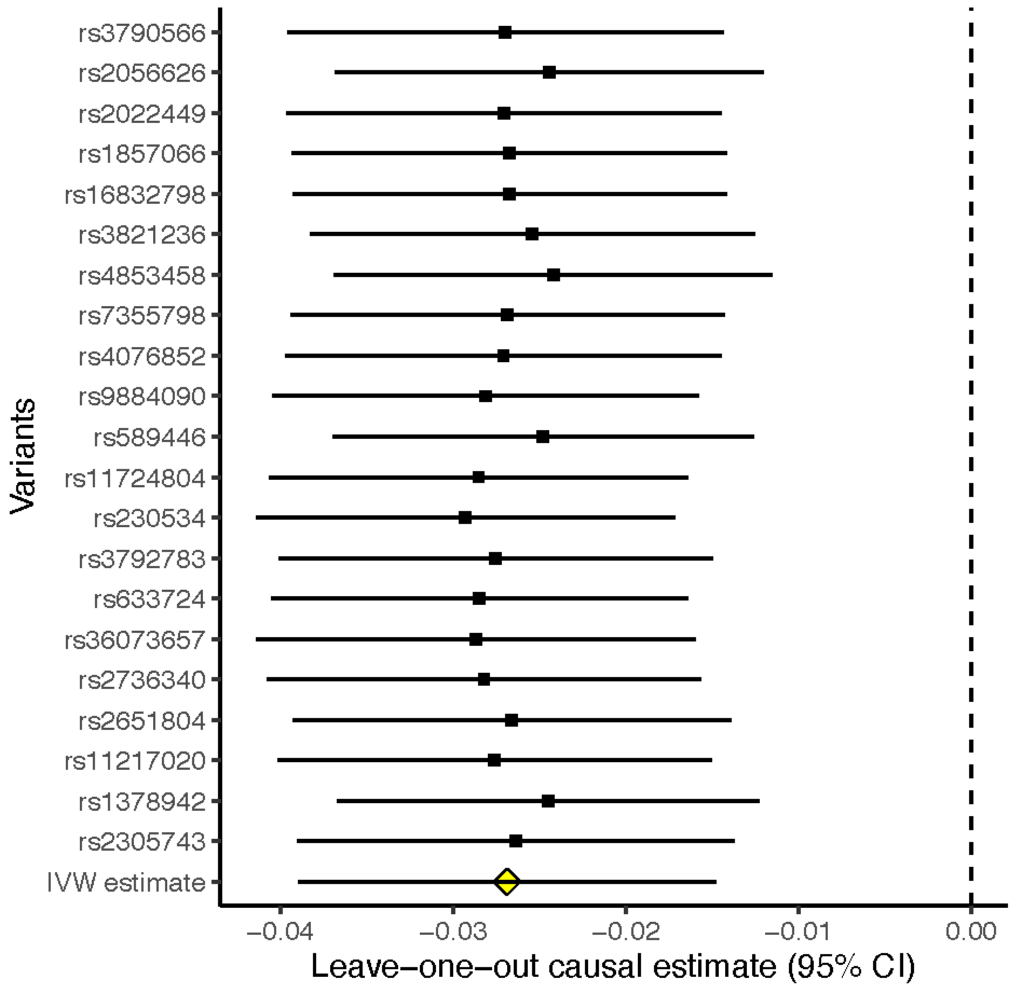

Figure S4 Leave-one-out sensitivity analysis for reverse MR (SSc to adiponectin)

References

1. Bowden J, Davey Smith G, Haycock PC, Burgess S. Consistent estimation in Mendelian randomization with some invalid instruments using a weighted median estimator. *Genetic epidemiology*. 2016;40:304-14.

2. Bowden J, Davey Smith G, Burgess S. Mendelian randomization with invalid instruments: effect estimation and bias detection through Egger regression. *International journal of epidemiology*. 2015;44:512-25.

3. Burgess S, Bowden J, Fall T, Ingelsson E, Thompson SG. Sensitivity analyses for robust causal inference from Mendelian randomization analyses with multiple genetic variants. *Epidemiology*. 2017;28:30-42.

4. Harrison PW, Amode MR, Austine-Orimoloye O, et al. Ensembl 2024. *Nucleic Acids Research*. 2023;52:D891-D9.
